# A national study of burnout, psychosocial work environment, and moral distress among neurosurgical doctors in Denmark

**DOI:** 10.1007/s00701-025-06468-w

**Published:** 2025-02-24

**Authors:** Thorbjørn Søren Rønn Jensen, Jakob Hakon, Markus Harboe Olsen, Helga Angela Gulisano, Tina Obbekjær, Frantz Rom Poulsen, Tiit Illimar Mathiesen

**Affiliations:** 1 Department of Neurosurgery, The Neuroscience Centre, Copenhagen University Hospital Rigshospitalet, Inge Lehmanns Vej 6, 2100 Copenhagen, Rigshospitalet Denmark; 2 Department of Neurosurgery, Clinical Institute and BRIDGE (Brain Research - Inter Disciplinary Guided Excellence), Odense University Hospital, University of Southern Denmark, DK-5000 Odense, Denmark; 3 Department of Neuroanesthesiology, The Neuroscience Centre, Copenhagen University Hospital, Rigshospitalet, Copenhagen, Denmark; 4 Department of Anaesthesiology, Zealand University Hospital, Køge, Denmark; 5 Department of Neurosurgery, Aalborg University Hospital, Aalborg, Denmark; 6 Department of Neurosurgery, Aarhus University Hospital, Aarhus, Denmark; 7 Department of Clinical Medicine, University of Copenhagen, Copenhagen, Denmark; 8 Department of Clinical Neuroscience, Karolinska Institutet, Stockholm, Sweden

**Keywords:** Copenhagen burnout inventory, Danish psychosocial work environment questionnaire, Response rate

## Abstract

**Background:**

Burnout is a condition of mental, emotional, and physical enervation affecting personnel working in human services and has been reported high among neurosurgical doctors. However, previous burnout reports are based on low response rates and measured by the Maslach Burnout inventory, which for several reasons has proven problematic. Burnout has not previously been investigated among neurosurgical doctors in Denmark. With this study we measure the prevalence of burnout among neurosurgical doctors in Denmark with sustainable methodology and a clinically relevant burnout interpretation.

**Methods:**

Burnout was measured among neurosurgical doctors in Denmark using the Copenhagen Burnout Inventory (CBI) consisting of three subscales measuring personal burnout, work-related burnout and patient-related burnout. To gain better understanding of factors contributing to burnout, the psychosocial working conditions and moral distress was measured using the Danish Psychosocial Work Environment Questionnaire (DPQ) and a questionnaire of eight items previously used to assess moral distress.

**Results:**

With 73 responders and a response rate of 90.1%, clinically relevant burnout was reported in 27.4% in personal burnout, 16.5% in work-related burnout and 5.5% in patient-related burnout. Cohort members with children living at home experienced a significant higher degree of burnout regarding work-related burnout and patient-related burnout.

Within the DPQ domains of ‘Demands at work’ and ‘Work organization and job content’, several moderate to strong correlations were observed between specific sub-dimensions and both personal and work-related burnout. Higher levels of the domain ‘Interpersonal relations’ was moderately correlated with lower levels of both personal and work-related burnout. In the testing of moral distress, only 2 responders (2.7%) scored as ‘somewhat injured’.

**Conclusion:**

Neurosurgical doctors in Denmark report relatively low prevalence of clinically relevant burnout. However, doctors with children living at home exhibited higher levels of work- and patient-related burnout. Our findings highlight the psychosocial work environment as a significant factor contributing to burnout, while moral distress appears to have a limited impact on the development of burnout in the study population.

**Supplementary Information:**

The online version contains supplementary material available at 10.1007/s00701-025-06468-w.

## Background

Burnout is a work-related condition of mental, emotional and physical enervation [37]. It is classified as a work-related rather than medical condition in the newly revised international classification of diseases (ICD)−11 [5]. Personnel in human services, especially health care workers, are especially vulnerable to developing this condition due to substantial time in intense commitment with other people [24]. Burnout may lead to increased risk of medical errors with a potential compromise in patient safety, and, personally, burnout is associated with negative individual psychological consequences including cynicism, exhaustion, and, if left unrecognized, development of depression [4, 22, 50]. Moreover, high degree of burnout among medical doctors’ increases health care costs, from low productivity, absenteeism, and the migration of affected doctors to other professions [3, 11, 25, 34, 37–39, 45, 49].

Recently, high levels of burnout were reported among neurosurgeons [13, 35]. Neurosurgery is regarded as an attractive and rewarding field, both in the general population and among medical doctors [25]. However, neurosurgery can be stressful and may possess several risk-factors of burnout, including a threatened work-life balance, a continuously high weekly workload and out-of-hours emergency coverage [44, 49]. As such, the reported prevalence of work-related burnout was recognized as high as 50% among neurosurgeons and neurosurgical trainees with only one-third of neurosurgical doctors recommending a career in neurosurgery [19, 25, 34, 50]. As neurosurgical burnout studies mainly originates from North America and Great Britain, it remains to be established if these observations are generalizable for hospital practice in those cultural environments only or if they reflect general qualities of how neurosurgery is practiced [3, 24, 25, 34, 39, 50]. Furthermore, previous burnout studies primarily utilized web-based questionnaires with frequently reported low response rates [34]. Web-based questionnaires often fail to control for multiple responses and suffer from other methodological confounders. Therefore, not only external but also internal validity of previous research is questionable.

With this study, we aim to investigate the prevalence of burnout among neurosurgical doctors in Denmark with sustainable methodology to achieve sufficient response rates and a clinically relevant interpretation of burnout. To gain better understanding of factors contributing to burnout among neurosurgeons, we further examined psychosocial working conditions and moral distress, which have not previously been thoroughly investigated.

The aim is to analyze if previously reported high burnout levels among neurosurgeons are reproducible among Danish neurosurgeons and to assess potential impact of confounding methodology.

## Methods

### Background and study population

This study included all senior and junior doctors employed at the four neurosurgical departments in Denmark: Aalborg University Hospital, Aarhus University Hospital, Odense University Hospital and Copenhagen University Hospital – Rigshospitalet. Aalborg University Hospital, Aarhus University Hospital and Odense University Hospital manages both spinal, peripheral nerve and cranial neurosurgery whereas Copenhagen University Hospital – Rigshospitalet solely manages cranial neurosurgery and peripheral nerve surgery. Consent for inclusion was obtained from all study participants.

In Denmark, neurosurgery is an independent specialty and the neurosurgical training last at least 5 years. The neurosurgical education requires a one-year internship followed by a four-year fixed residency. The first permanent position as a neurosurgical specialist is as a junior attending. A permanent position as an attending can be applied at earliest after 5 years as a junior attending. Dependent on the neurosurgical department, the on-call service is managed either by a neurosurgical intern, resident or junior attending as first in line with either a chief resident, junior attending or attending as second in line as back-up. In case of the first line on-call service being management by intern or resident, either a chief resident or junior attending is on-call from home.

In Denmark, 37 h is the standard working week with 5 mandatory fully paid vacation weeks and an additional voluntary paid vacation week every year. There is paid overtime, additional medical education, pension, paid sick leave, and paternity/maternity leave. Though, as neurosurgery is the most popular medical specialty in Denmark based on yearly applications per resident position, it is well-known that the applicates spend free time gaining neurosurgical expertise. The Danish health care system is fully taxfinanced and, consequently, the neurosurgical service is free for all patients and supplementary private health insurance is not necessary.

### Measurement questionnaires

Burnout was measured using the Copenhagen Burnout Inventory (CBI) [18]. CBI consists of three subscales measuring personal burnout, work-related burnout and patient-related burnout and is validated in the accuracy of burnout identification in populations within the human service sector [9, 18, 47]. Each subscale ranges from 0 to 100, with a higher score indicating a higher degree of burnout (supplementary Fig. 3a and b). Burnout severity was categorized in accordance with Møller CM et al. Scores less than 25 were categorized as ‘no symptoms of burnout’, scores between 25 and 49.9 as ‘light burnout’, scores between 50 and 74.9 as ‘moderate burnout’ (symptoms of burnout that should be dealt with), and scores of 75 or more as ‘severe burnout’ (severe symptoms of burnout that must be dealt with immediately) [27].

Following the measurement of burnout, further understanding of the work environment was attained by using the Danish Psychosocial Work Environment Questionnaire (DPQ) [8]. We included the following four domains: 1) Demands at work, 2) work organization and job content, 3) interpersonal relations: cooperation and leadership, and 4) Reactions to the work situation. The four domains included a total of 31 different psychosocial work environment dimensions, all items within each dimension were included in the questionnaire.

As moral distress may be a central factor for doctors working in the neurosurgical field, an assessment of this factor was included in this study. Moral distress is defined as a psychological effect a healthcare provider experience when experiencing inadequacy to sustain professional values due to external limitations [28]. The limitations may be caused by interpersonal, administrative, or legal barriers reducing the healthcare workers capability to act consistently with professional values [17]. Moral distress was measured through a questionnaire of eight items previously used to assess moral distress among neurosurgeons [10, 20]. Each item is scored using a Likert scale (0–4) in two dimensions (frequency and intensity). Item scores are multiplied and then summed to yield composite score of 0–128, with 128 indicating the highest level of moral distress.

### Inclusion and exclusion

This study was conducted between November 1st and December 1st, 2023. This period was chosen as it does not conflict with national holidays or similar mandatory clinical low activity periods. The questionnaires were anonymously filled out on paper, placed in a neutral, closed envelopes and collected by a local secretary at each department without thorough study knowledge. To assuredly obtain anonymized data, sex was not part of the demographics. All the envelopes across Denmark were assembled by an independent person without knowledge of the doctors working in neurosurgery in Denmark and the questionnaire were recorded into a REDCap database from where a statistician could access the data. Only aggregated data was presented for the research group.

At the neurosurgical departments in Denmark, neurologist in-training have a shorter stay as part of their specialization. These doctors were excluded from the study. Furthermore, we excluded neurosurgical doctors employed at the department within three months before the study. Also, professors, chief attendings and members of this research group were excluded.

### Ethics

The study was approved by the Danish Data Protection Agency (p-2023–14576) and conducted in accordance with the Declaration of Helsinki.

### Statistics

Data was entered into the Research Electronic Data Capture (REDCap) and analysed using R version 4.2.1 (R Core Team, Vienna, Austria). Continuous data is presented as mean and 95% confidence interval (CI) or median and interquartile range (IQR) depending on normality. Categorical data is presented using numbers and percentages. Any missingness is presented in detail. CBI was aggregated using the methodology described by Møller CM et al., Clausen T et al., and Epstein EG et al. [8, 10, 27].

To ascertain correlation magnitude between summary estimates Pearsons correlation coefficient was used [36]. A subgroup analysis was conducted regarding site, age, weakly working hours, side job, relationship, children living at home, and night job. To investigate subgroups differences, an interaction analysis using linear regression was used. If such an interaction was identified, pairwise t-tests were carried out to investigate differences. The p-value in the pairwise t-tests were Bonferroni corrected.

## Results

Of the invited 81 doctors from Aalborg University hospital, Aarhus University Hospital, University Hospital of Southern Denmark and Copenhagen University Hospital – Rigshospitalet, 73 neurosurgical doctors responded to the questionnaire resulting in a response rate of 90.1%. Of the 73 responders, 35.6% were younger than 40 years, 17.8% were above 55 years and the majority of 45.2% were between 40 and 55 years. Regarding the medical positions, 42.5% were attendings, 17.8% were junior attendings, 6.8% were chief residents, and 30.1% were residents. Full cohort demographics including missing data are presented in Table 1.

**Table 1:** Demographic and work-related characteristics of neurosurgical doctors in Denmark

	Overall *n* = 73
Age (%)	
< 40 years	26 (35.6)
> 55 years	13 (17.8)
40–55 years	33 (45.2)
Missing	1 (1.4)
Position (%)	
Attending	31 (42.5%)
Chief resident	5 (6.8%)
Junior attending	13 (17.8%)
Resident	22 (30.1%)
Missing	2 (2.7%)
Average time at work per week (%)	
< 41 h	21 (28.8%)
> 49 h	13 (17.8%)
41–49 h	35 (47.9%)
Missing	4 (5.5%)
Average number of night shifts per month	
Median [IQR]	4.00 [0.00;4.00]
Mean (95%CI)	2.84 (2.30;3.38)
Missing	16 (21.9%)
Average number of 24-h shifts per month	
Median [IQR]	2.00 [1.00;4.00]
Mean (95%CI)	2.34 (1.90;2.78)
Missing (%)	3 (4.1)
Scheduled time for administration (> 5 h per week)	8 (11.0%)
Scheduled time for research/lecturer work (> 5 h per week)	11 (15.1%)
Side job	42 (57.5%)
Number of hours per week on side job	
Median [IQR]	5.25 [3.00;8.00]
Mean (95%CI)	5.36 (4.32;6.40)
Missing (%)	31 (42.5%)
Permanent relationship/cohabitation	60 (82.2%)
Children living at home	47 (64.4%)

### Burnout amongst neurosurgical doctors in Denmark

**Fig. 1:**
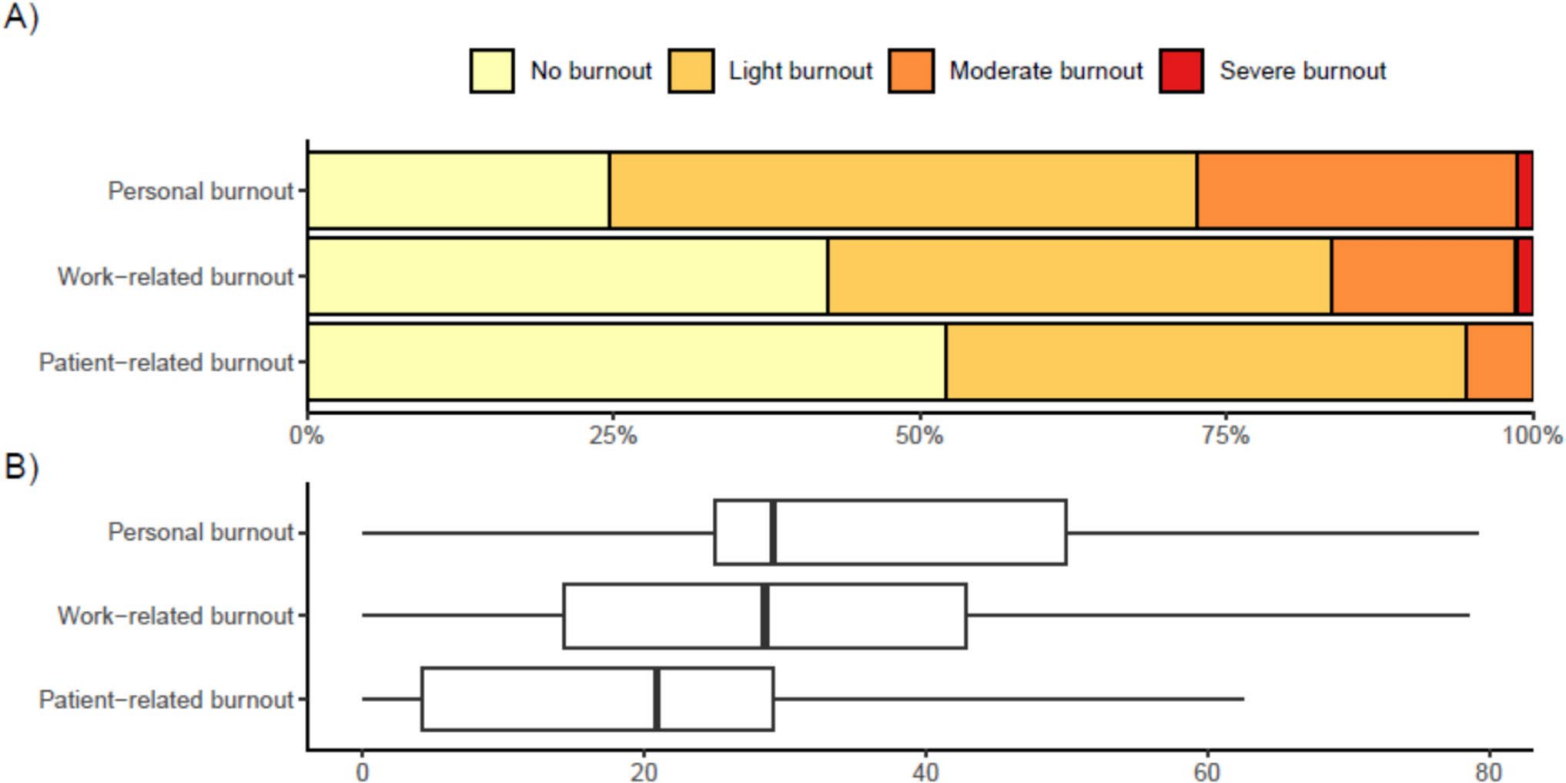
Severity of the burnout subtypes amongst neurosurgical doctors in Denmark. **A** Division of burnout into ‘no burnout’, ‘light burnout’, ‘moderate burnout’ and ‘severe burnout’. **B** Joint median burnout score for each burnout subtype

The mean (95%CI) burnout scores were 35.37 (31.20;39.53) for personal burnout, 28.77 (24.73;32.80) for work-related burnout and 20.95 (17.22;24.67) patient-related burnout. Of the burnout subtypes, 18 (24.7%) reported no personal burnout, 35 (47.9%) light personal burnout, 19 (26%) moderate personal burnout and 1 (1.4%) severe personal burnout. Regarding work-related burnout, 31 (42.5%) experienced no burnout, 30 (41.1%) light burnout, 11 (15.1%) moderate burnout and 1 (1.4%) severe burnout. Of patient-related burnout, 38 (52.1%) reported no burnout, 31 (42.5%) light burnout and 4 (5.5%) moderate burnout (Fig. 1 and Table 2).

**Table 2:** Median and mean levels of burnout subtypes

	Overall *N* = 73
Personal burnout	
• Median [IQR]	29.17 [25.00;50.00]
• Mean (95%CI)	35.37 (31.20;39.53)
Personal burnout – group (%)	
• Light burnout	35 (47.9)
• Moderate burnout	19 (26.0)
• No burnout	18 (24.7)
• Severe burnout	1 (1.4)
Work-related burnout	
• Median [IQR]	28.57 [14.29;42.86]
• Mean (95%CI)	28.77 (24.73;32.80)
Work-related burnout – group (%)	
• Light burnout	30 (41.1)
• Moderate burnout	11 (15.1)
• No burnout	31 (42.5)
• Severe burnout	1 (1.4)
Patient-related burnout	
• Median [IQR]	20.83 [4.17;29.17]
• Mean (95%CI)	20.95 (17.22;24.67)
Patient-related burnout – group (%)	
• Light burnout	31 (42.5)
• Moderate burnout	4 (5.5)
• No burnout	38 (52.1)

*IQR* Interquartile range, *CI* Confidence interval

**Fig. 2:**
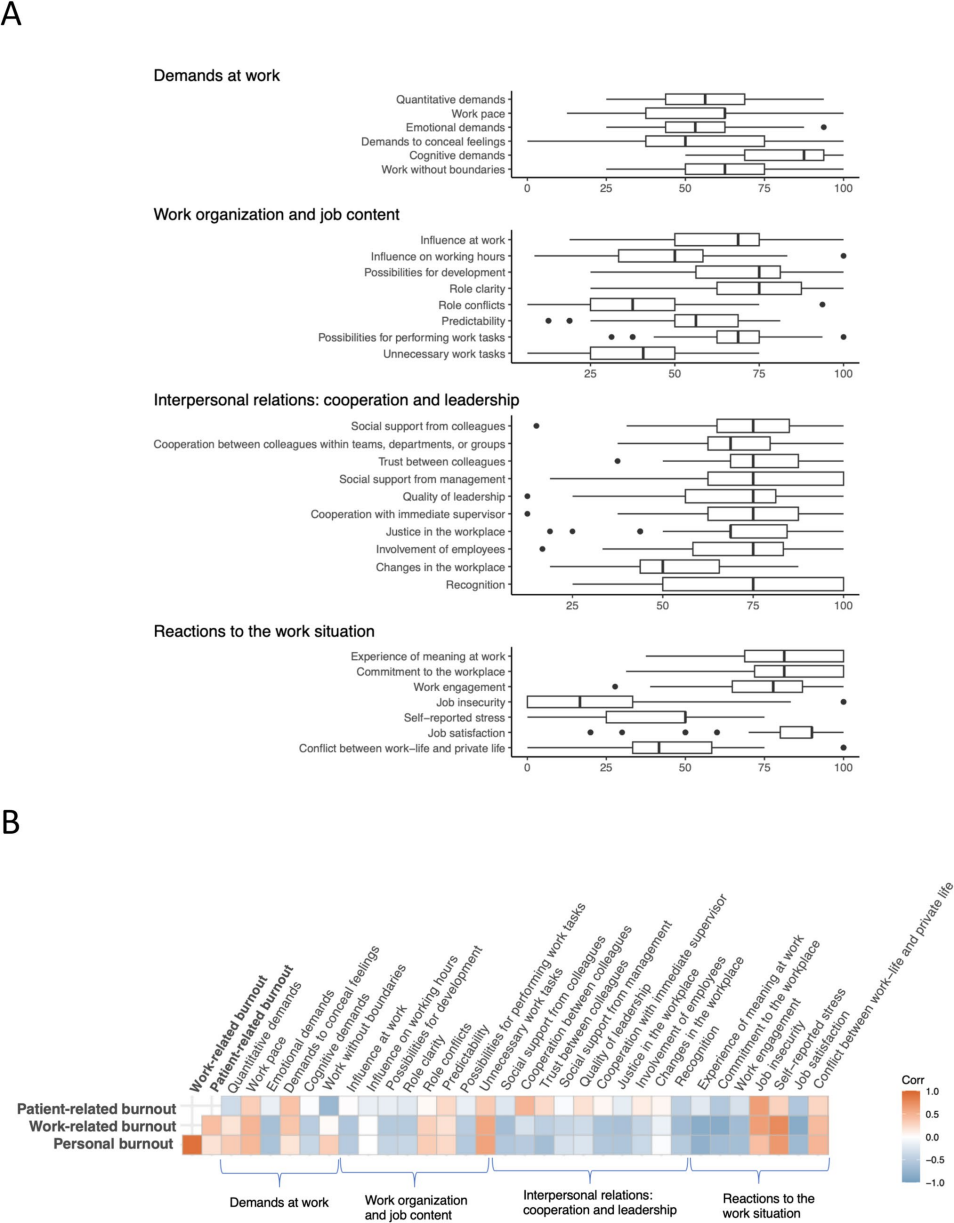
**A** Medians of the dimensions within the Danish Psychosocial Work Environment Questionnaire (DPQ). Individual questions within each dimension ranges from very low extent (0), to low extend (25), to somewhat (50), to large extend (75), to very large extend (100). **B** Heat map demonstrating the correlation between the three burnout subtypes and dimension means within the DPQ

Focusing on only the burnout levels requiring intervention, which is moderate and severe burnout, 20 (27.4%) doctors reported personal burnout, 12 (16.5%) doctors reported work-related burnout, and 4 (5.5%) doctors reported patient-related burnout. We observed a strong correlation between personal burnout and work-related burnout (R = 0.93). In contrast, the correlation between personal burnout and patient-related burnout was weak (R = 0.19; Fig. 2A and B).

In a subgroup analysis, a comparison of burnout between the different cohort demographics and work-related characteristics of neurosurgical doctors in Denmark was performed (Table 1). Differences in burnout between cohort members with and without children living at home was statistically significant regarding work-related burnout and patient-related burnout. Work-related burnout was 31.2% (26.2;36.2) for cohort members with children living at home and 22.3% (16.7;27.9) for cohort members without children living at home (*p* = 0.027). Concerning patient-related burnout the mean burnout was 23.8% (19.4;28.3) for cohort members with children living at home vs 14.7% (8.1;21.2) without children living at home (*p* = 0.018). There was no statistically significant difference for personal burnout between cohorts with and without children living at home. We found no statistical difference in burnout when comparing the other cohort demographics after Bonferroni correction including burnout between the neurosurgical centers (Supplementary Fig. 1A-G).

### Danish psychosocial work environment questionnaire

Regarding the DPQ variables, personal and work-related burnout exhibited moderate to strong correlations with several dimensions, whereas patient-related burnout generally demonstrated weak correlations with the DPQ variables (Fig. 2A and B). Within the DPQ domains ‘Demands at work’ and ‘Work organization and job content’, we identified moderate to strong positive correlations between the burnout subtypes (personal and work-related) and DPQ dimensions. These dimensions include ‘Work pace’ (R = 0.47 for personal burnout; R = 0.48 for work-related burnout) and ‘Unnecessary work tasks’ (R = 0.60 for personal burnout; R = 0.68 for work-related burnout). Within the domain ‘Reactions to the work situation’ moderate to strong positive correlations were found between the burnout subtypes (personal and work-related) and the following dimensions: ‘Conflicts between work-life balance’ (R = 0.48 for personal burnout; R = 0.44 for work-related burnout), ‘Self-reported stress’ (R = 0.67 for personal burnout; R = 0.76 for work-related burnout) and ‘Job insecurity’ (R = 0.42 for personal burnout; R = 0.63 for work-related burnout), although the latter was generally reported to a low extent.

There were particularly strong inverse correlations within the domain’Reactions to the work situation’. Especially, ‘Work engagement’ was negatively correlated with personal burnout (R = −0.66) and work-related burnout (R = −0.68). ‘Commitment to the workplace’ also showed strong negative correlations with personal burnout (R = −0.68) and work-related burnout (R = −0.86). Similarly, ‘Experience of meaning at work’ was highly inversely correlated with personal burnout (R = −0.81) and work-related burnout (R = −0.85). ‘Job satisfaction’ demonstrated negative correlations of R = −0.71 with personal burnout and R = −0.86 with work-related burnout. The dimension ‘Strong interpersonal relations’ was generally moderately negatively corelated to personal and work-related burn-out (Fig. 2B). In the domain of ‘Demands at work and work organization’*,* several moderate correlations were identified. ‘Emotional demands’ were inversely correlated with personal burnout (R = −0.62) and work-related burnout (R = −0.64). ‘Influence at work’ also showed moderate negative correlations with personal burnout (R = −0.53) and work-related burnout (R = −0.58). Furthermore, the dimension ‘Possibility for development’ was moderately associated with reduced personal burnout (R = −0.43) and work-related burnout (R = −0.54).

### Moral distress

In the testing of moral distress, 69 cohort members (94.5%) were not morally injured. Only 2 cohort members (2.7%) scored as ‘somewhat injured’ (Table 3). All domains scored a low frequency of distress resulting in a very low composite score (Supplementary Fig. 2).

**Table 3:** Moral distress amongst neurosurgical doctors in Denmark

	Overall *N* = 73
Moral distress	
• Median [IQR]	4.00 [1.00;6.00]
• Mean (95%CI)	3.92 (3.13;4.71)
• Missing	2 (2.7%)
Morally injured	
• Not injured	69 (94.5%)
• Somewhat injured	2 (2.7%)
• Missing	2 (2.7%)

## Discussion

Burnout is a well-recognized problem in the general population and among health care personal [1, 2]. The condition has been investigated among nurses and doctors, and throughout medical specialties [2, 31, 33]. In this study burnout, the psychosocial work environment and moral distress were investigated among neurosurgical doctors in Denmark.

The concept of burnout may appear intuitive but needs clarification. ‘Burnout’ was initially described by Maslach and Jackson as a composite of emotional exhaustion, depersonalization, and reduced personal accomplishment [21, 23]. They constructed the “Maslach Burnout Inventory” (MBI) in order to identify and measure the condition [24]. The MBI-measure is somewhat problematic because of circularity and unclear use of how the aggregated composite could be interpreted [18]. Hence, a better tool was needed, which led to the construction of the CBI [18]. Kristensen et al. limit burnout symptoms to fatigue and exhaustion and differentiate between personal burnout, work-related burnout, and client related burnout. Available studies using MBI claim findings of burnout in 50–60% of normal populations of health care workers although that high proportion does not suffer from comparable morbidity in terms of decreased health [26, 32, 45, 48]. Larger studies with CBI also report burnout levels around 50% among health care workers, although the studies suffered from low response rates [15, 46]. It is important for a study to address levels of burnout that may constitute a health problem and require interventions; for CBI this level equals to burnout levels classified as moderate and severe [18, 46].

When translating the burnout rates in our study into severity levels for the two clinically related dimensions, 16.5% of respondents reported work-related burnout and 5.5% reported patient-related burnout at thresholds warranting intervention. Most cases fell within the moderate severity range, while only two doctors reported severe burnout. Furthermore, 27.4% of respondents reported moderate to severe levels of personal burnout, reflecting a general state of exhaustion and fatigue experienced by the individual. In general, we could not reproduce the high rates of burnout reported by previous studies of neurosurgeons [3, 25, 34, 39]. Using the CBI, Salloum et al. reported substantially higher levels of personal burnout (median 47% vs 35% in the current report) and work-related burnout (median 49% vs 29% in the current report) among neurosurgical trainees in the UK and Ireland. In contrast, levels of patient-related burnout were similar (median 19.6% vs 21% in the current report). Most previous studies used the MBI [3, 24, 25, 39]. MBI was earliest in use but was criticized for methodological problems including circularity and a confusing design with an aggregate composite. Also, the original MBI version defined burnout as a condition restricted to health-care workers [18], but the implementation of MBI in burnout literature reflects over 20 different applications [2, 3, 12, 25, 33, 47, 49]. CBI was constructed to solve the methodological issues and provide a more practical tool for burnout research, management, and, in contrast to MBI, CBI is freely available for use [18]. Recently, CBI has been favored in international studies from the 2020s [27, 31, 34, 47]. Results on burnout may agree between MBI and CBI [41], but unfortunately MBI and CBI can also show conflicting results when applied to measure burnout in similar cohorts, thus making a comparison potentially misleading [2]. Previous studies demonstrate joint burnout level measured by either MBI or CBI in health care personal and health care students between 31% to 79.9% [42, 43], which is higher than we found. The dichotomization between moderate/severe burnout and no/mild burnout is standard in literature on CBI [46]. We conclude that the burnout rates in our population are lower than previously reported and that the difference does not primarily reflect choice of questionnaire.

Importantly, a confounder that has not been sufficiently addressed in previous studies is response rates. The response rates on neurosurgical populations were 12–42%, which limits generalizability of results severely (7–13, 31, 32). Moreover, previous researchers failed to include sensitivity analyses. It is conceivable that invited responders who were indifferent to burnout were less motivated to respond or that potentially exhausted invitees were unable to. Therefore, reports may be confounded by falsely negative or positive generalizations from a cohort motivated to respond to the questionnaires. Our study had a 90.1% response rate which secures internal validity for the population of Danish neurosurgeons. One study that is comparable in terms of response rates is a Danish study on vascular surgeons [27]. Møller CM et al. investigate CBI measured burnout among vascular surgeons and vascular surgeons in training in Denmark [27]. Vascular surgery has similarities to neurosurgery including high-risk procedures, a stressful environment, critically ill patients, and several weakly working hours spend on-call. A viable comparison is therefore possible between neurosurgical and vascular doctors in Denmark. With an acceptable response rate of 82%, Møller et al. found that 16 and 4% of vascular doctors in Denmark reported burnout at levels requiring intervention within work-related and patient-related burnout. The figures are almost identical to 16.5 and 5.5% that experienced burnout requiring intervention in our neurosurgical cohort. Regardless of external validity of previous studies, our and Møller CM et al.’s Danish figures must be considered comparatively low. It is probable that local culture and national conditions affect nation-wide burnout levels. Government controlled factors such as planned weakly working hours, possibilities of sick leaves, vacation, further education, pension, salary, paternity/maternity varies between countries, and may influence the risk of burnout. In Denmark, the overall working conditions is considerably good including lower weakly working hours compared to other European and North American countries. Work-life balance is a prioritized issue in Danish society and Denmark scores consistently among the top happiest countries in the world [16].

Although we found lower levels than expected based om previous global experience, up to a fifth of our responders reported burnout at levels that require interventions. The kind of stress that associates with burnout appears to vary over time [18], but a conflict of duties and values appears to be an important cause of burnout. Work-life balance and ethical stress belong to areas of potential conflict as is a conflict of duties to patients or duties to family. In analogy to our data on the Danish neurosurgeons, Salloum et al. found that children living at home correlated with an increased burnout score [34]. However, the effect of home-living children on burnout is not consistent. Some studies present a protective effect especially against depersonalization, though these studies measure burnout by MBI [6] [7]. Therefore, the difference effect from home-living children on burnout could again reflect the difference in measurement tools. Theoretically, both scenarios may be explained as home-living children may contribute to the conflict between duties to the family and the work, but as a protective factor, doctors with home-living children have been proposed to leave work both physically and mentally earlier focusing their thoughts on topics outside work. Furthermore, it has been suggested that doctors with home-living children spend more leisure time with their kids, which leads to more positive activities to compensate for work-related burnout.

### Danish psychosocial work environment questionnaire

Regarding the DPQ variables, patient-related burnout demonstrated only weak associations, whereas personal and work-related burnout exhibited moderate to strong correlations within several dimensions. In the domains of ‘Demands at work’ and ‘Work organization’, positive correlations emerged for dimensions such as ‘Work pace’ and ‘Unnecessary work tasks’. Furthermore, ‘Emotional demands’ and ‘Influence at work’ were inversely correlated with burnout, with moderate effects. Within the domain ‘Reaction to the work situation’ inverse correlations were found for dimensions such as ‘Work engagement’, ‘Commitment to the workplace’, ‘Experience of meaning at work’ and ‘Job satisfaction’. Additionally, most dimensions within ‘Interpersonal relations’ had moderate negative correlations with personal and work-related burnout. These findings align to some extent with those of Møller CM et al., who identified strong associations between personal and work-related burnout and psychosocial factors, particularly in the domains of ‘Work organization’ and ‘Interpersonal relationships’. However, they found weaker associations with ‘Demands at work’, which contrasts with the findings of the present study. Notably, in both studies, patient-related burnout was less pronounced compared to personal and work-related burnout. This indicates that the psychosocial work environment, rather than patient care itself, may serve as a key contributor to burnout.

### Moral distress

Moral distress was assessed using a validated moral distress survey, revealing a low frequency of distress across all domains, resulting in a very low composite score. Our findings suggest that the surgeons evaluated in this study generally experienced minimal nonmedical pressure to perform aggressive surgeries or provide care for patients without clear treatment goals. We therefore conclude that moral distress has a limited impact on the development of burnout in the study population.

### Strength and limitations

As described, burnout can be measured by different questionnaires, which have shown to cause concurrent or divergent results [2]. The comparison of burnout between studies using the CBI and the MBI has thus proven difficult [2]. We were able to identify studies investigating burnout in neurosurgical doctors using CBI, but several burnout studies measured burnout by MBI and a fully comparison of our study to the published literature is difficult [19, 24, 25, 27, 29, 34, 42, 44]. Lately, CBI appears to be the favored tool to measure burnout [27, 31, 34, 47].

It is likely that demographic factors other than those included in this study may impact burnout. The selected demographical factors were based on similar burnout studies [27], but we recognize that other factors may influence burnout such as the specific personality for each doctor, number and age of the home-living children, employment status of the cohabitant and the subspecialization of the neurosurgical doctor. Furthermore, we anonymized sex to achieve a higher response rate, though this factor has shown importance in other studies [30].

A noticeable strength of this study is the high response rate of 90.1% which secures a high degree of internal validity. To achieve cohort representativeness in a survey a response rate of minimum 80% must be reached [14]. In previous studies it has been shown that a face-to-face approach to subject recruitment are superior to recruitment by email or internet surveys [40]. We believe that the methodological approach by filling out questionnaires on paper in this study increased the response rate, which may be an inspirational practice in upcoming studies using questionnaires.

## Conclusion

Burnout amongst neurosurgical doctors in Denmark is relatively low. Doctors with children living at home had a higher degree of work-related and patient-related burnout. We cannot make direct comparisons to published literature since previous questionnaire-studies on burnout are probably confounded by low response rates. Our data are internally valid and appear to be externally valid for Danish surgeons also of other specialties. Future questionnaire-based studies must consider response rates and, with a response rate of 90.1% achieved in study, we propose the use of physical questionnaires to secure representative responses.

## Supplementary Information

Below is the link to the electronic supplementary material.

Supplementary Material 1 (DOCX 331 KB)

## Data Availability

No datasets were generated or analysed during the current study.
