# Supplementary material for: A national study of burnout, psychosocial work environment, and moral distress among neurosurgical doctors in Denmark
Source: Acta Neurochir (Wien). 2025 Feb 24;167(1):53. doi: 10.1007/s00701-025-06468-w (PMC11850451; doi:10.1007/s00701-025-06468-w)
Supplement: Supplementary file 1 — Supplementary Material 1 (DOCX 331 KB) [file 701_2025_6468_MOESM1_ESM.docx]

**Supplementary material**

**A**


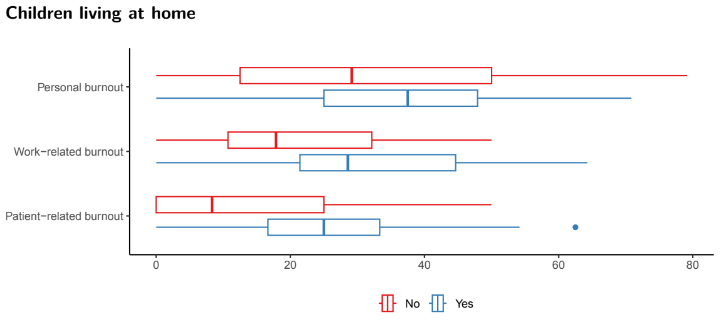


**B**

**
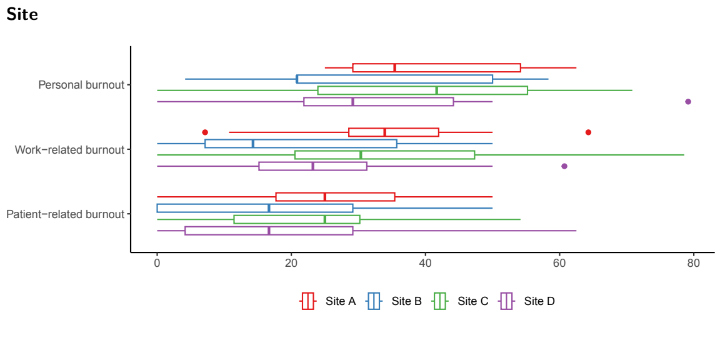
**

**C**


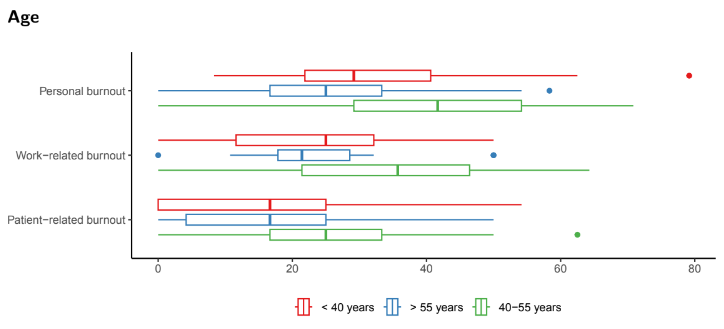


**D
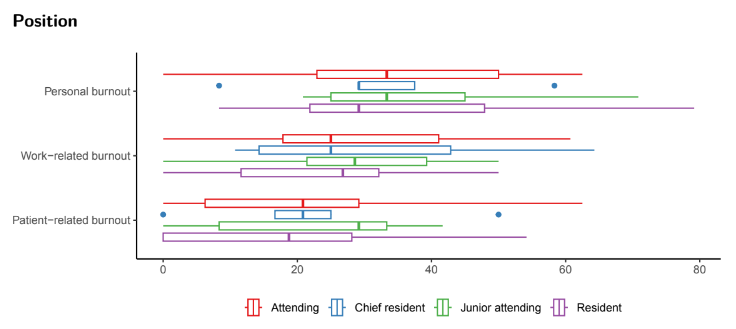
**

**E
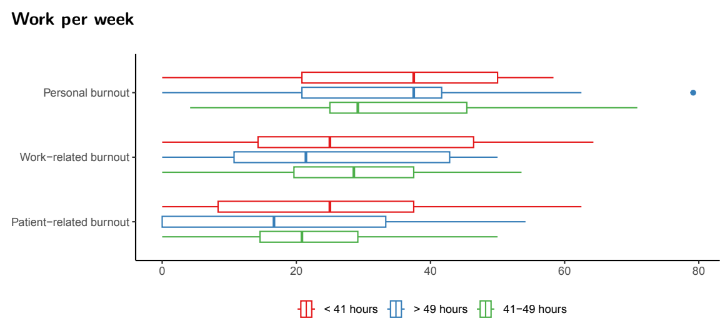
**

**F
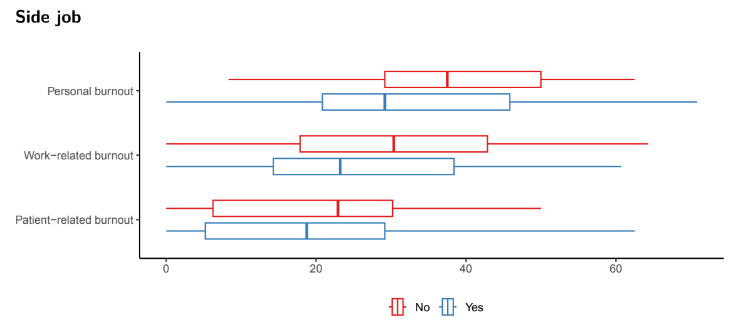
**

**G
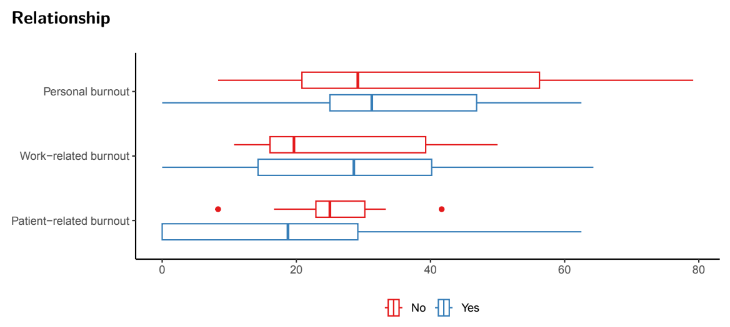
**

**Supplementary figure 1A-G.** Boxplots for subgroup analysis of burnout subtypes scores compared between children living at home, neurosurgical sites, age, position, work hours per week, side job and relationship. Presented values are medians. Only the subgroup analysis of children living at home was statistically significant for work-related and patient-related burnout.

1. P-values for the comparison of burnout subtypes between children living at home: Personal burnout: p=0.10, work-related burnout: p=0.02, patient-related burnout: p=0.01.
2. P-values for the comparison of burnout between neurosurgical centers: Personal burnout: p=0.23, work-related: p=0.03 (With Bonferroni-correction; site A vs B: p=0.08, site A vs C: p=1.0, site A vs D: p=0.72, site B vs C: p=0.06, site B vs D: p=1.0, site C vs D: p=0.62), patient-related burnout: p=0.80. Site A: Aarhus University Hospital (n=18, response rate 81%), Site B: Aalborg University Hospital (n=13, response rate 92%), Site C: Odense University Hospital (n=20; response rate 100%), Site D: Copenhagen University Hospital – Rigshospitalet (n=22, response rate 92%). Site A: Aarhus University Hospital, Site B: Aalborg University Hospital, Site C: Odense University Hospital, Site D: Copenhagen University Hospital – Rigshospitalet.
3. P-values for the comparison of burnout subtypes between age groups: Personal burnout: p=0.03 (With Bonferroni-correction; >55 years vs < 40 years: p=1.0, 40-55 years vs < 40 years: p=0.15, 40-55 years vs >55 years: p=0.07), work-related burnout: p=0.09, patient-related burnout: p=0.16.
4. P-values for the comparison of burnout subtypes between positions: Personal burnout: p=0.93, work-related burnout: p=0.85, patient-related burnout: p=0.82.
5. P-values for the comparison of burnout subtypes between work hours per week: Personal burnout: p=0.99, work-related burnout: p=0.70, patient-related burnout: p=0.29.
6. P-values for the comparison of burnout subtypes between side job: Personal burnout: p=0.07, work-related burnout: p=0.36, patient-related burnout: p=1.0.
7. P-values for the comparison of burnout subtypes between relationship: personal burnout p=0.43, work-related burnout: p=0.81, patient-related burnout: p=0.23.


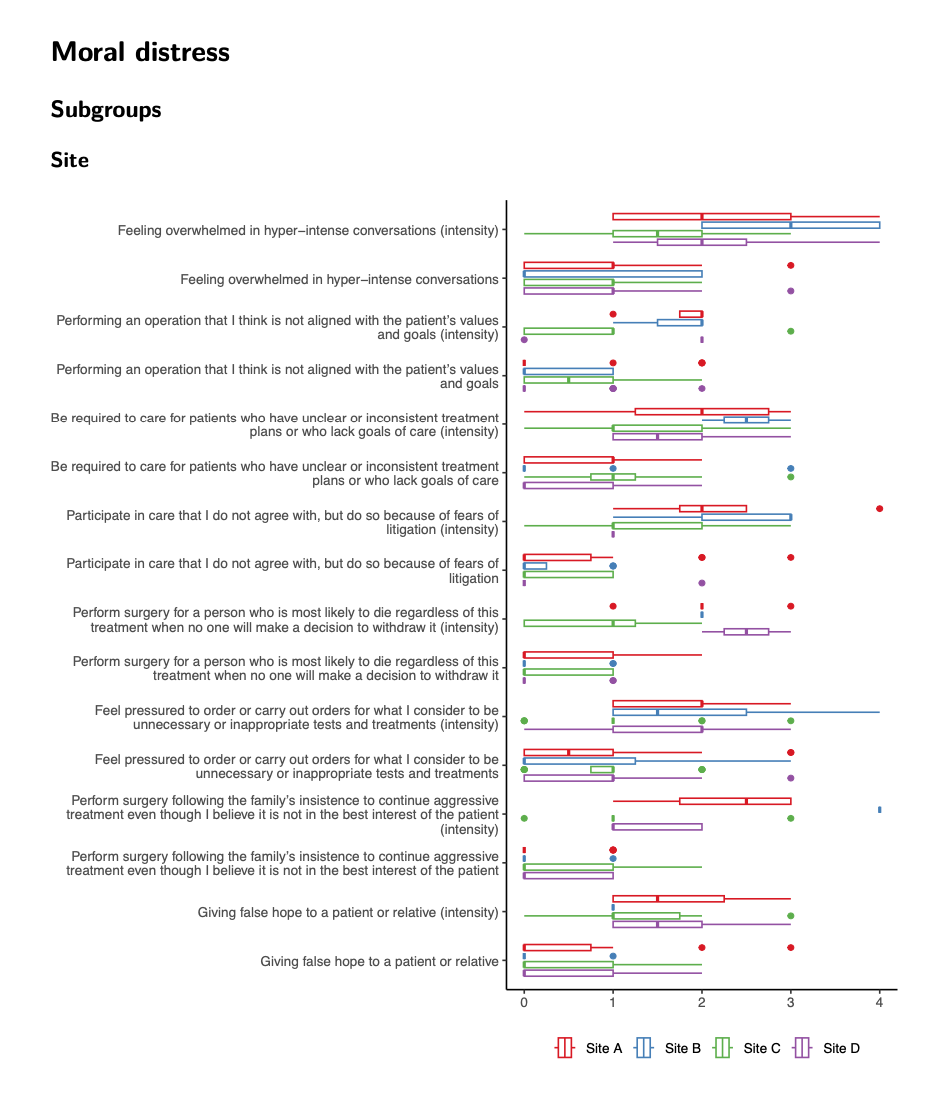


**Supplementary figure 2**. Measurement of moral distress divided between sites. Moral distress was measured for eight items using a Likert scale (0-4) in two dimensions (frequency and intensity). All areas of moral distress scored a low frequency of distress resulting in a very low composite score.

Site A: Aarhus University Hospital, Site B: Aalborg University Hospital, Site C: Odense University Hospital, Site D: Copenhagen University Hospital – Rigshospitalet.

| **Demographical questions, supplementary figure 3a** | | | | | | | | | | | |
| --- | --- | --- | --- | --- | --- | --- | --- | --- | --- | --- | --- |
| Age | | | | < 40 years | | 40-55 years | | | | >55 years | |
| Position | Resident | | Chief resident | | | Junior attending | | | | Attending | |
| Average time at work per week | | <37 hours | | | 37-40 hours | | 41-49 hours | | | >49 hours | |
| Average number of night shifts per month (write number) | | | | | | | | |  | | |
| Average number of 24-hour shifts per month (write number) | | | | | | | | |  | | |
| Scheduled time for administration (>5 hours per week) | | | | | | | | | Yes | | No |
| Scheduled time for research/lecturer work (>5 hours per week) | | | | | | | | | Yes | | No |
| Side job | | | | | | | | Yes | | No | |
| Number of hours per week on side job (write number) | | | | | | | |  | | | |
| Permanent relationship/cohabitation | | | | | | | | Yes | | No | |
| Children living at home | | | | | | | | Yes | | No | |

| **Copenhagen Burnout Inventory**  *Put one mark for each question, and answer all questions* | | | | | |
| --- | --- | --- | --- | --- | --- |
| **Personal burnout** | *Always* | *Often* | *Sometimes* | *Seldom* | *Never/almost never* |
| How often do you feel tired? | □ | □ | □ | □ | □ |
| How often are you physically exhausted? | □ | □ | □ | □ | □ |
| How often are you emotionally exhausted? | □ | □ | □ | □ | □ |
| How often do you think: ”I can’t take it anymore”? | □ | □ | □ | □ | □ |
| How often do you feel worn out? | □ | □ | □ | □ | □ |
| How often do you feel weak and susceptible to illness? | □ | □ | □ | □ | □ |
| **Work-related burnout** | *To a very high degree* | *To a high degree* | *Somewhat* | *To a low degree* | *To a very low degree* |
| Is your work emotionally exhausting? | □ | □ | □ | □ | □ |
| Does your work frustrate you? | □ | □ | □ | □ | □ |
| Do you feel burnt out because of your work? | □ | □ | □ | □ | □ |
|  | *Always* | *Often* | *Sometimes* | *Seldom* | *Never/almost never* |
| Do you feel that every working hour is tiring for you? | □ | □ | □ | □ | □ |
| Are you exhausted in the morning at the thought of another day at work? | □ | □ | □ | □ | □ |
| Do you feel worn out at the end of the working day? | □ | □ | □ | □ | □ |
|  | *Never/almost never* | *Seldom* | *Sometimes* | *Often* | *Always* |
| Do you have enough energy for family and friends during leisure time? | □ | □ | □ | □ | □ |

| **Copenhagen Burnout Inventory**  *Put one mark for each question, and answer all questions* | | | | | |
| --- | --- | --- | --- | --- | --- |
| **Patient-related burnout** | *To a very high degree* | *To a high degree* | *Somewhat* | *To a low degree* | *To a very low degree* |
| Do you find it hard to work with patients? | □ | □ | □ | □ | □ |
| Does it drain your energy to work with patients? | □ | □ | □ | □ | □ |
| Do you find it frustrating to work with patients? | □ | □ | □ | □ | □ |
| Do you feel that you give more than you get back when you work with patients? | □ | □ | □ | □ | □ |
|  | *Always* | *Often* | *Sometimes* | *Seldom* | *Never/almost never* |
| Are you tired of working with patients? | □ | □ | □ | □ | □ |
| Do you sometimes wonder how long you will be able to continue working with patients? | □ | □ | □ | □ | □ |
| Are you exhausted in the morning at the thought of another day at work? | □ | □ | □ | □ | □ |

| **Demografiske spørgsmål (sæt kryds), supplementary figure 3b** | | | | | | | | | | | | | |
| --- | --- | --- | --- | --- | --- | --- | --- | --- | --- | --- | --- | --- | --- |
| Alder | | | | | < 40 år | | | 40-55 år | | | >55 år | | |
| Stilling | Reservelæge | | 1.reservelæge | | | Afdelingslæge | | | | | Overlæge | | |
| Gennemsnitlig arbejdstid per uge | | <37 timer | | 37-40 timer | | | 41-49 timer | | | | >49 timer | | |
| Gennemsnitlig antal nattevagter per måned *(skriv antal)* | | | | | | | | | |  | | | |
| Gennemsnitlig antal døgnvagter per måned *(skriv antal)* | | | | | | | | | |  | | | |
| Skemasat tid til administration *(>5 timer ugentligt)* | | | | | | | | | | Ja | | | Nej |
| Skemasat tid til forskning/lektorarbejde *(>5 timer ugentligt)* | | | | | | | | | | Ja | | | Nej |
| Har du et bijob | | | | | | | | | Ja | | | Nej | |
| Antal timer ugentlig på bijob? *(skriv antal)* | | | | | | | | |  | | | | |
| Har du et fast forhold/samlever? | | | | | | | | | Ja | | | Nej | |
| Har du hjemmeboende børn | | | | | | | | | Ja | | | Nej | |

| **Copenhagen Burnout Inventory**  *Sæt kun ét kryds ud for hvert spørgsmål, og besvar alle spørgsmålene* | | | | | |
| --- | --- | --- | --- | --- | --- |
| **Personlig udbrændthed** | *Altid* | *Ofte* | *Sommetider* | *Sjældent* | *Aldrig/næsten aldrig* |
| Hvor tit føler du dig træt? | □ | □ | □ | □ | □ |
| Hvor tit er du fysisk udmattet? | □ | □ | □ | □ | □ |
| Hvor tit er du følelsesmæssigt udmattet? | □ | □ | □ | □ | □ |
| Hvor tit tænker du: ”nu kan jeg ikke klare mere?” | □ | □ | □ | □ | □ |
| Hvor tit føler du dig udkørt? | □ | □ | □ | □ | □ |
| Hvor tit føler du dig svag og modtagelig overfor sygdom? | □ | □ | □ | □ | □ |
| **Arbejdsrelateret udbrændthed** | *I meget høj grad* | *I høj grad* | *Delvist* | *I ringe grad* | *I meget ringe grad* |
| Udmatter dit arbejde dig følelsesmæssigt? | □ | □ | □ | □ | □ |
| Føler du dig frustreret af dig arbejde? | □ | □ | □ | □ | □ |
| Føler du dig udbrændt på grund af dit arbejde? | □ | □ | □ | □ | □ |
|  | *Altid* | *Ofte* | *Sommetider* | *Sjældent* | *Aldrig/næsten aldrig* |
| Føler du, at hver time er en belastning for dig når du er på arbejde? | □ | □ | □ | □ | □ |
| Er du udmattet om morgenen ved tanken om endnu en dag på arbejdet? | □ | □ | □ | □ | □ |
| Føler du dig udkørt når din arbejdsdag er slut? | □ | □ | □ | □ | □ |
|  | *Aldrig/næsten aldrig* | *Sjældent* | *Sommetider* | *Ofte* | *Altid* |
| Har du overskud til at være sammen med familie og venner i fritiden? | □ | □ | □ | □ | □ |

| **Copenhagen Burnout Inventory**  *Sæt kun ét kryds ud for hvert spørgsmål, og besvar alle spørgsmålene* | | | | | |
| --- | --- | --- | --- | --- | --- |
| **Patientrelateret udbrændthed** | *I meget høj grad* | *I høj grad* | *Delvist* | *I ringe grad* | *I meget ringe grad* |
| Føler du at det er en belastning at arbejde med patienter? | □ | □ | □ | □ | □ |
| Bliver du tappet for energi af at arbejde med patienter? | □ | □ | □ | □ | □ |
| Føler du at det er frustrerende at arbejde med patienter? | □ | □ | □ | □ | □ |
| Føler du at du giver mere end du får igen i dit arbejde med patienter? | □ | □ | □ | □ | □ |
|  | *Altid* | *Ofte* | *Sommetider* | *Sjældent* | *Aldrig/næsten aldrig* |
| Er du træt af at arbejde med patienter? | □ | □ | □ | □ | □ |
| Er du sommetider i tvivl om hvor længe du orker at blive ved med at arbejde med patienter? | □ | □ | □ | □ | □ |
| Føler du dig udkørt når din arbejdsdag er slut? | □ | □ | □ | □ | □ |

**Supplementary figure 3a and 3b.** The Copenhagen Burnout Inventory in an translated English version (3a) and the original Danish version used in this study (3b).
